# Correlations of cognitive function with serum levels of homocysteine, sex hormone binding globulin, and leptin in patients with schizophrenia

**DOI:** 10.12669/pjms.40.10.8923

**Published:** 2024-11

**Authors:** Yunqiang Xu, Xiulian Qian, Minqiao Zhang, Ming Liang

**Affiliations:** 1 Yunqiang Xu, Department of Psychiatry, Xiangshan County Hospital of Traditional Chinese Medicine, Medical and Health Group Third Hospital, Ningbo 315700, Zhejiang, China; 2 Xiulian Qian Department of Psychiatry, The Third Hospital of Quzhou, Ningbo 315700, Zhejiang, China; 3 Minqiao Zhang, Department of Psychiatry, Xiangshan County Hospital of Traditional Chinese Medicine, Medical and Health Group Third Hospital, Ningbo 315700, Zhejiang, China; 4 Ming Liang, Department of Psychiatry, Xiangshan County Hospital of Traditional Chinese Medicine, Medical and Health Group Third Hospital, Ningbo 315700, Zhejiang, China

**Keywords:** Homocysteine, Sex hormone binding globulin, Leptin level, Schizophrenia, Cognitive function

## Abstract

**Objective:**

To investigate the correlations of cognitive function with serum levels of homocysteine (Hcy), sex hormone binding globulin (SHBG), and leptin in patients with schizophrenia.

**Methods:**

This was retrospective study. This study included 58 patients with schizophrenia (SCZ) as observation group and 60 clinically healthy individuals as control group admitted to Xiangshan County Hospital of Traditional Chinese Medicine Medical and Health Group Third Hospital from March 2022 to June 2023. Serum levels of Hcy, SHBG, and leptin in the two groups were measured, and Pearson correlation analysis was conducted to investigate the correlations between the Hcy, SHBG, and leptin levels and the psychiatric symptoms and cognitive function in the observation group.

**Results:**

The Hcy and SHBG levels in the observation group were significantly higher than those in the control group (*p<*0.05, respectively), and the leptin level was lower than that in the control group (*p<*0.05). The observation group scored significantly lower than the control group for each dimension of the MCCB (*p<*0.05, respectively). In the observation group, Hcy was positively correlated with negative symptoms and the PANSS total score (*p<*0.05, respectively) and was negatively correlated with scores for the leptin was negatively correlated with negative symptoms and the PANSS total score (*p<*0.05, respectively) and was positively correlated with scores for speed of processing (SOP), WMS-III, BVMT, and MSCEIT (*p<*0.05, respectively).

**Conclusions:**

Increased levels of Hcy and SHBG and decreased leptin are strongly associated with the occurrence of cognitive impairment in SCZ cases. Therefore, clinical observation and monitoring of these markers can help identify changes in patient conditions.

## INTRODUCTION

Schizophrenia(SCZ) is a clinically common severe mental disorder with symptoms such as delusions and hallucinations, which have devastating impacts on a patient’s social function and quality of life.^1^ Reportedly, patients with SCZ often show some extent of cognitive impairment, typically involving verbal and visual-spatial working memories, and the mental disability rate is up to 80%.^2^,^3^ This kind of cognitive impairment plays a significant role in the conversion of mental disorders and thus is considered predictive of the occurrence and prognosis of SCZ in susceptible individuals.^4^,^5^ Homocysteine(Hcy), a sulfur-containing amino acid, can induce neuronal apoptosis at an elevated level and is closely related to the occurrence of nervous system disorders and cognitive impairment.^6^ Sex hormone binding globulin (SHBG), a member of the steroid-binding globulin family, can regulate the levels of estrogen and testosterone in the body, thereby exerting an indirect influence on the central nervous system function.^7^

Leptin, an adipose-derived hormone, acts as an important player in glucose and lipid metabolism and has recently been found to have a close relationship with the emotions and behavior of patients with SCZ.^8^ In light of these findings, this study aimed to provide new evidence for the clinical diagnosis and treatment of SCZ by analyzing the correlations between cognitive function and serum levels of Hcy, SHBG, and leptin in patients with the disease.

## METHODS

This was a retrospective study which included 58 patients with schizophrenia (SCZ) as observation group and 60 clinically healthy individuals as control group admitted to Xiangshan County Hospital of Traditional Chinese Medicine Medical and Health Group Third Hospital from March 2022 to June 2023. Data were retrieved from the hospital information and management system. Collected their various information of all patients, including main clinical features, laboratory tests, etc. The observation group had 24 males and 34 females aged between 25 and 60 years, with an average age of (39.12±9.22) years. The control group had 28 males and 32 females of 24 to 60 years old, and the average age was (38.88±9.12) years. The two groups lacked statistically significant differences in general information (all *p>* 0.05) and thus were considered comparable.

### Ethical Approval:

The study was approved by the Institutional Ethics Committee of Xiangshan County Hospital of Traditional Chinese Medicine Medical and Health Group Third Hospital (No.:2020020; March 12, 2020), and written informed consent was obtained from all participants.

### Inclusion criteria:

- Meeting the diagnostic criteria for SCZ.^9^
- Not using any medication that might affect cognitive function during the previous month.
- Having primary or higher education levels.

### Exclusion criteria:

- Severe nervous system disorders.
- Organic brain disorders.
- Incapable of effective communication or full participation.
- Pregnant or breastfeeding.

### Psychiatric symptoms:

The Positive and Negative Syndrome Scale (*P*ANSS)^10^ with 30 items in total was adopted to assess the psychiatric symptoms of the observation group. The scale is composed of three subscales: the positive, negative, and general psychopathology scales. The positive scale covers seven aspects, including delusions, conceptual disorganization, hallucinations, excitement, grandiosity, suspiciousness/persecution, and hostility; the negative scale contains seven items, such as blunted affect, emotional withdrawal, poor rapport, passive/apathetic social withdrawal, difficulty in abstract thinking, lack of spontaneity, and stereotyped thinking; the general psychopathology scale encompasses 16 items, namely somatic concern, anxiety, guilt feelings, tension, mannerisms/posturing, depression, motor retardation, uncooperativeness, unusual thought content, disorientation, poor attention, lack of judgment and insight, disturbance of volition, poor impulse control, preoccupation, and active social avoidance. Higher PANSS scores indicate more severe symptoms. The Cronbach’s alpha is 0.832.

The MATRICS™ Consensus Cognitive Battery (MCCB)^11^ was used for cognitive function evaluation in both groups. The MCCB includes 10 tests: Trail Making Test, Category Fluency, Symbol Coding (BACS), Continuous Performance Test-Identical Pairs (CPT-IP), Letter-Number Span, Spatial Span (the Wechsler Memory Scale–Third Edition, WMS-III), verbal learning (Hopkins Verbal Learning Test, HVLT), visual learning (Brief Visuospatial Memory Test, BVMT), Mazes (Neuropsychological Assessment Battery, NAB), and emotion management (Mayer-Salovey-Caruso Emotional Intelligence Test, MSCEIT™). These tests primarily measure seven cognitive domains: speed of processing (SOP), attention/vigilance (CPT-IP), working memory (WMS-III), verbal learning and memory (HVLT), visual learning and memory (BVMT), reasoning and problem solving (NAB), and social cognition (MSCEIT™). The primary raw score on each dimension is computed to yield an equivalent standard deviation (SD) of (50±10), with a higher score indicating better cognitive function. The scale has a Cronbach’s α value of 0.863.

Fasting venous blood (five mL) was drawn from every participant and injected into a blood collection tube. After standing for 30 minutes, serum was isolated from each blood sample by centrifugation at 3,500 rpm for 20 minutes and then stored in a refrigerator. The BK-600 automatic biochemical analyzer (Biobase Bioindustry (Shandong) Co., Ltd.) was used for measuring Hcy, SHBG, and leptin in the two groups via enzyme-linked immunosorbent assay (ELISA). The assay kits were purchased from Beijing Jianping Jiuxing Biology Medicine Technology Co., Ltd. and were used exactly as instructed by the manufacturer.

### Statistical Analysis:

The software SPSS 23.0 was used for data processing. Enumeration data were expressed as n% and examined using the chi-square test. Measurement data were presented as (x±s), and intergroup comparisons were analyzed using the t-test. Pearson correlation analysis was performed to investigate the correlations between the Hcy, SHBG, and leptin levels and the psychiatric symptoms and cognitive function of patients with SCZ. P<0.05 indicated a difference of statistical significance.

## RESULTS

The observation group consisting of 58 patients scored (19.26±2.47) for positive symptoms, (22.34±3.27) for negative symptoms, and (32.74±4.58) for general psychopathology. The PANSS total score was (74.34±7.45).

Compared with the control group, the observation group had lower scores for SOP, CPT-IP, WMS-III, HVLT, BVMT, NAB, and MSCEIT (all *p<* 0.05). Table-I. The observation group had higher levels of Hcy and SHBG (*p<* 0.05, respectively) and a lower level of leptin compared with the control group. Table-II.

**Table-I:** Intergroup comparison of cognitive function scores (*χ̅*±*S*).

Group	n	SOP	CPT-IP	WMS-III	HVLT	BVMT	NAB	MSCEIT
SCZ	58	16.36±2.12	24.38±3.02	23.36±2.30	24.31±3.12	21.29±2.35	22.19±2.77	24.36±3.08
Control	60	28.32±2.62	35.12±2.21	32.40±2.38	31.62±3.27	27.82±3.74	28.38±2.46	32.22±3.52
*t-value*		27.209	22.096	20.965	12.406	11.286	12.847	12.883
*P-value* ^△^		0.000	0.000	0.000	0.000	0.000	0.000	0.000

***Notes:*** ^△^independent-sample t-test.

**Table-II:** Intergroup comparison of Hcy, SHBG, and leptin levels (*χ̅*±*S*).

Group	n	Hcy (μmol/L)	SHBG (ng/mL)	Leptin (μg/L)
SCZ	58	22.39±5.31	269.75±8.13	7.69±1.76
Control	60	9.21±2.69	101.37±8.70	11.36±1.80
*t-value*		17.084	108.536	11.174
*P-value* ^△^		0.000	0.000	0.000

***Notes:*** ^△^independent-sample t-test.

Pearson correlation analysis showed that in the observation group, Hcy was positively correlated with negative symptoms and the PANSS total score (*p<* 0.05, respectively); SHBG was positively correlated with negative symptoms and the PANSS total score (*p<* 0.05, respectively); leptin was negatively correlated with negative symptoms and the PANSS total score (*p<* 0.05, respectively). Table-III.

**Table-III:** Correlations of psychiatric symptoms with Hcy, SHBG, and leptin levels in the observation group.

Marker	Hcy (μmol/L)		SHBG (ng/mL)		leptin (μg/L)	

	r	p	r	p	r	p
Positive symptoms	-0.041	0.762	0.031	0.817	-0.022	0.870
Negative symptoms	0.147	0.022	0.044	0.043	0.216	0.014
General psychopathology	0.173	0.194	0.203	0.127	0.123	0.359
PANSS total score	0.157	0.039	0.154	0.048	0.163	0.022

Pearson correlation analysis showed that in the observation group, Hcy was negatively correlated with the SOP, CPT-IP, and BVMT scores (*p<* 0.05, respectively); SHBG was negatively correlated with the SOP, CPT-IP, and BVMT scores (*p<* 0.05, respectively); leptin was positively correlated with the SOP, CPT-IP, and BVMT scores (*p<* 0.05, respectively). Table-IV.

**Table-IV:** Correlations of cognitive function with Hcy, SHBG, and leptin levels in the observation group.

Marker	Hcy (μmol/L)		SHBG (ng/mL)		Leptin (μg/L)	

	r	p	r	p	r	p
SOP	-0.153	0.253	-0.084	0.530	-0.189	0.015
CPT-IP	-0.168	0.027	-0.099	0.046	-0.083	0.537
WMS-III	-0.148	0.028	-0.082	0.538	-0.167	0.021
HVLT	-0.019	0.887	0.012	0.929	-0.119	0.373
BVMT	-0.114	0.033	-0.060	0.002	-0.238	0.043
NAB	-0.233	0.039	-0.140	0.295	-0.154	0.249
MSCEIT	-0.094	0.482	-0.051	0.004	-0.159	0.033

## DISCUSSION

The results of this study showed that compared with the control group, the observation group received lower scores in many domains of cognitive function, adding evidence for varying degrees of cognitive decline in patients with SCZ. Hcy, an intermediate product of the methionine cycle, can bind with adenosine and produce S-adenosyl-homocysteine to reduce methylation. An increase in the Hcy level can induce neuronal apoptosis and entail a higher risk of cognitive impairment.^12^ In this study, the Hcy level in the observation group was higher than in the control group and was positively correlated with negative symptoms and the PANSS total score, while negatively correlated with cognitive function scores. These findings indicate a strong association between cognitive decline and increased Hcy in patients with SCZ. Abnormal metabolism of Hcy, a cytotoxic, sulfur-containing amino acid, can lead to central neurotransmitter disorders, which has been widely reported to have a close association with depression and Alzheimer’s disease.^13^,^14^ This excitatory neurotoxic substance has a direct or indirect impact on the glutamatergic system of the central nervous system, promoting the synthesis of endogenous nitric oxide, lowering the activity of its synthase, and thereby suppressing neural development and normal neural network construction.^15^

As people are living a hectic modern life under growing social pressure, SCZ has a constantly rising incidence, which is reported to reach about 1.0%.^16^ In most cases, SCZ is manifested through negative symptoms such as anxiety, apathy, and lack of interest, which are believed to compromise neurological and cognitive functions.^17^,^18^ Bora et al.^19^ proposed that an overlap of susceptible genes exists between SCZ and cognitive impairment. With cognitive impairment being a typical yet easily overlooked core symptom of the disease, about 80% of the susceptible population experiences changes in thinking and behavioral patterns before such symptoms meet the diagnostic criteria.^20^ As the disease progresses, many patients tend to have symptom fluctuations, suffer from a progressive course, and even develop suicidal thoughts.^21^ Therefore, it is of vital importance to research cognitive function-related mechanisms on the molecular level, thereby achieving an early diagnosis, effective patient condition evaluation, and better prognosis.

The present study also revealed that the SHBG level in the observation group was higher than in the control group and had positive correlations with psychiatric symptoms yet negative correlations with the cognitive function scores. All these are evidence for the close relationship between an elevated SHBG level and cognitive impairment in patients with SCZ. SHBG, a steroid-binding globulin, is chiefly derived from the liver. It plays a role in regulating estrogen levels and, to some extent, is involved in insulin metabolism as it can aggravate glucose metabolism disorders and cause indirect functional damage to the central nervous system. Xu et al.^22^ discovered that an elevated SHBG level means an increased risk of cognitive decline in patients with Alzheimer’s disease.

In addition, the high-affinity SHBG can bind to corticotropin-releasing hormone binding protein (CRHBP), a key player in synaptic and neuronal signal transduction, resulting in reduced bioavailability and functional compromise of the nervous system.^23^ Last but not least, this study showed that the leptin level in the observation group was lower than in the control group; this marker was negatively correlated with negative symptoms and the PANSS total score and was positively correlated with cognitive function scores. The study results suggest that a decreased level of leptin is potentially associated with cognitive impairment in patients with SCZ. Leptin, a single-chain protein, can induce oxidative stress and aggravate endothelial cell damage.^24^

On the other hand, leptin works synergistically with insulin-like growth factors to promote body growth and metabolism, and its receptor is present in the hypothalamus and hippocampus.^25^ Hamilton et al.^26^ viewed that leptin has a profound impact on hippocampus functions and is involved in a series of major cellular changes in learning and memory; In terms of learning and cognitive function, leptin is believed to enhance cognitive function and provide neuroprotective benefits. Therefore, a lower level of leptin may indicate poor cognitive function.

### Limitations of this study:

It includes small sample size, small course of treatment, and short follow-up time. In addition, only the clinical efficacy was observed in this study, which led to the observation indexes being subjectively influenced by patients and the indexes being difficult to quantify, so further studies are still needed to improve them in the future.

## CONCLUSIONS

When cognitive impairment occurs, patients with SCZ seem to have significantly increased Hcy and SHBG and reduced leptin. These changes are associated with psychiatric symptoms and cognitive function. Clinically, these serological markers may reflect changes in the condition of patients with SCZ.

### Authors’ Contributions:

**YX** and **XQ:** Designed this study and prepared this manuscript, and were responsible and accountable for the accuracy or integrity of the work. **MZ and ML:** Performed the statistical analysis, Review and participated in its design. All authors read and approved the final manuscript.
